# Nitrous oxide-induced funicular myelosis and polyneuropathy: a case report with follow-up MR imaging

**DOI:** 10.1007/s10072-023-06660-9

**Published:** 2023-03-16

**Authors:** Natalie Gancarczyk, Asadeh Lakghomi, Oliver Kaut

**Affiliations:** 1 Department of Neurology, University Hospital of Bonn, Bonn, Germany; 2 Department of Neuroradiology, University Hospital of Bonn, Bonn, Germany

**Keywords:** Nitrous oxide, Vitamin B12, B12 deficiency, Methylmalonic acid, Funicular myelosis, Toxic neuropathy

The COVID-19 pandemic has led to a shift in drug use to legal substances. In particular, there has been an increase in the use of nitrous oxide (N_2_O), which can cause neurological and psychiatric disorders [1].

We report a case of a 22-year-old male with no previous illness who presented to our emergency department with a 1-week history of new neurological symptoms. It began with tingling and numbness involving both feet and all fingertips, gradually rising until below the chest as well as both arms with a disorder of fine motor skills of the hands and erectile dysfunction. The initial suspected diagnosis was Guillain-Barré syndrome.

On examination, he showed fine motor function impairments in both hands, and rope, heel, and toe walking were not possible. Both the Romberg sign and the Lhermitte sign were positive. He had a tingling sensation from dermatome Th4 downwards and on the upper limbs. Vibration at the lateral malleolus and patella and sensation of joint position at the tips of the big toes were affected bilaterally. Reflexes at the knees occurred only after the Jendrassik maneuver, whereas deep tendon reflexes at the upper extremity and Achilles tendon were absent.

Blood tests were performed to rule out the most common causes of polyneuropathy and myelopathy. CSF examination was inconspicuous.

Neurography revealed sensorimotor axonal demyelinating polyneuropathy, while nerve ultrasound was unremarkable. Magnetic resonance imaging of the cervical spine showed the typical findings of funicular myelosis (Fig. 1a, b).

**Fig. 1:**
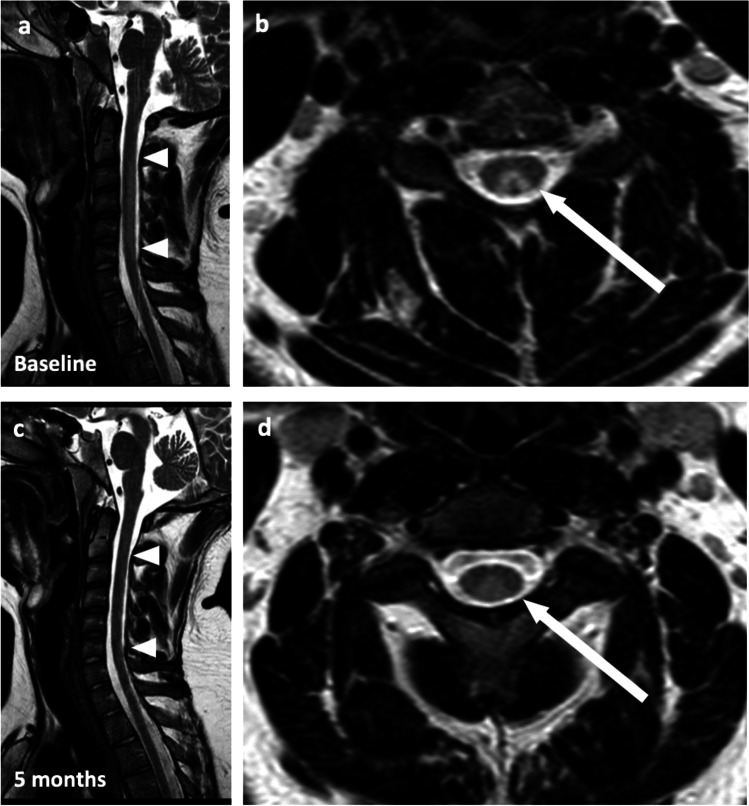
**a**–**d** T2-weighted sagittal mDixon sequences and axial TSE sequences of the cervical myelon. The baseline shows sagittal dorsal long-range signal enhancements (cervical vertebra C2-6; triangle) of the myelon (**a**). In the axial (**b**) sequences, they are prominent in the posterior strands (“V-shaped,” arrow). After 5 months of vitamin B12 substitution, lesions (arrow) have regressed (**d**). At cervical vertebra C2-6, a residual is found with remaining flat signal elevations (triangle) of the dorsal spinal cord section (**c**)

The patient had been taking N_2_O regularly for about 2–3 months. Shortly before the beginning of symptoms, he had consumed higher doses.

N_2_O irreversibly oxidizes the cobalt ion of vitamin B12, rendering it non-functional, leading to decreased conversion of homocysteine to methionine, which results in increased homocysteine levels and demyelination due to diminished myelin production [2, 3]. It also results in the inability of methylmalonyl-CoA mutase to function, resulting in higher methylmalonic acid (MMA) levels [2].

In this case, the vitamin B12 serum level was within the normal range. Of note, MMA was significantly elevated at 5494 nmol/l (reference 50–300), as was homocysteine at 99.37 μmol/l (reference 3.2–10.7).

We initiated an intramuscular vitamin B12 substitution with 1000 μg once daily started on the day of admission for a total of 7 days, followed by oral supplementation. Meanwhile, the MMA level rapidly decreased to normal (Fig. 2).

**Fig. 2:**
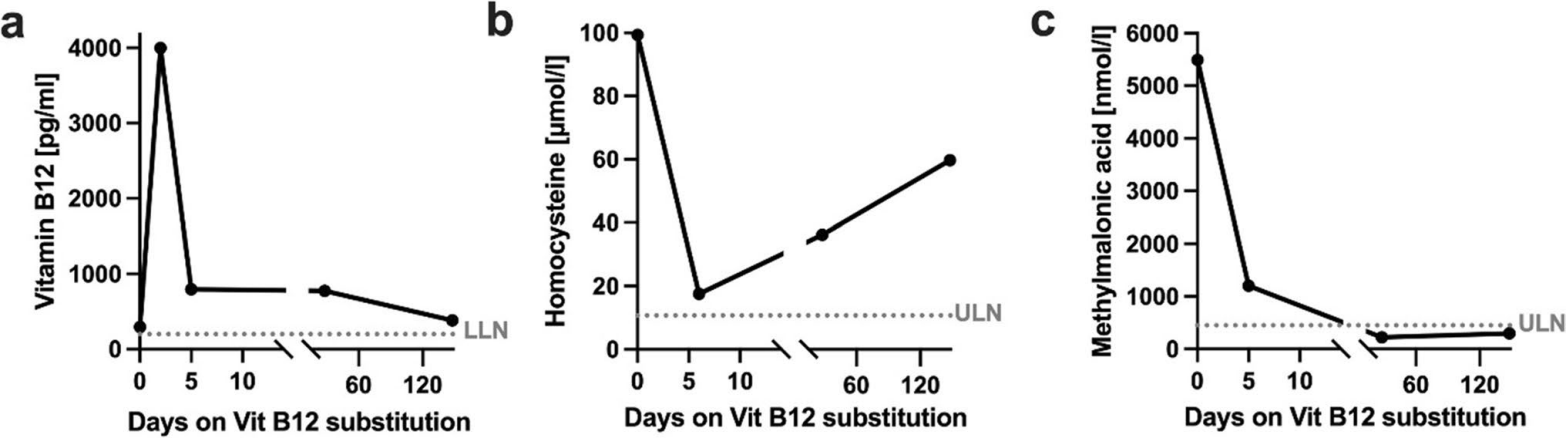
Course of the laboratory parameters vitamin B12 (**a**), homocysteine (**b**), and methylmalonic acid (**c**) at initial presentation and during the course of vitamin B12 substitution

At the time of discharge, tingling paresthesia had regressed, and upper extremity reflexes had returned. Neurography showed improvement, but latencies of cortical somatosensory-evoked potentials from both legs were prolonged.

At follow-up 1 and 5 months after hospitalization, further improvement was noted, motor impairments were no longer present, and tingling only affected the fingertips and toes. Positional sense at the big toes and vibratory sensation at the lateral malleolus improved significantly. Lower extremity reflexes had returned, and tightrope and toe walking were feasible.

After 5 months, imaging showed significant regression (Fig. 1c, d), but neurography showed a slight worsening of findings, while mild fine motor dysfunction returned.

Our case illustrates the importance of taking a detailed medical history, as patients may conceal unpleasant facts. If there is a typical history and clinical course, homocysteine and MMA should be determined in addition to vitamin B12 levels, as serum levels may be normal in functional vitamin B12 deficiency [1, 3].

## Data Availability

The datasets used during this case report are available from the corresponding author on reasonable request.
